# Human Genomic Loci Important in Common Infectious Diseases: Role of High-Throughput Sequencing and Genome-Wide Association Studies

**DOI:** 10.1155/2018/1875217

**Published:** 2018-03-20

**Authors:** Gerald Mboowa, Ivan Sserwadda, Marion Amujal, Norah Namatovu

**Affiliations:** ^1^Department of Immunology and Molecular Biology, College of Health Sciences, Makerere University, P.O. Box 7072, Kampala, Uganda; ^2^Department of Medical Microbiology, College of Health Sciences, Makerere University, P.O. Box 7072, Kampala, Uganda

## Abstract

HIV/AIDS, tuberculosis (TB), and malaria are 3 major global public health threats that undermine development in many resource-poor settings. Recently, the notion that positive selection during epidemics or longer periods of exposure to common infectious diseases may have had a major effect in modifying the constitution of the human genome is being interrogated at a large scale in many populations around the world. This positive selection from infectious diseases increases power to detect associations in genome-wide association studies (GWASs). High-throughput sequencing (HTS) has transformed both the management of infectious diseases and continues to enable large-scale functional characterization of host resistance/susceptibility alleles and loci; a paradigm shift from single candidate gene studies. Application of genome sequencing technologies and genomics has enabled us to interrogate the host-pathogen interface for improving human health. Human populations are constantly locked in evolutionary arms races with pathogens; therefore, identification of common infectious disease-associated genomic variants/markers is important in therapeutic, vaccine development, and screening susceptible individuals in a population. This review describes a range of host-pathogen genomic loci that have been associated with disease susceptibility and resistant patterns in the era of HTS. We further highlight potential opportunities for these genetic markers.

## 1. Introduction

HIV/AIDS, tuberculosis, and malaria are 3 major global public health threats causing substantial morbidity, mortality, negative socioeconomic impact, and human suffering [1]. Whole-genome sequencing (WGS) of hosts and their cognate pathogens has transformed our understanding of the contribution of genomics in infectious disease processes. As the antibiotic era changed our understanding of infections so has the genomic revolution. The host-pathogen coevolutionary “arms race” is a phenomenon that has been described to result from interaction within host innate, adaptive immune responses, exposure to antibiotics, and competition with commensal microbiota [2, 3]. When positive selection in one geographical region causes large allele frequency differences between populations than those expected for neutrally evolving alleles, high frequency of the derived allele (i.e., when a new allele increases to a frequency higher than that expected under genetic drift) [4] and HTS technologies are harnessed and used to decipher the nature and dynamics of these signals. The dynamics of host-pathogen interactions (e.g., length of exposure, geographical spread, morbidity, mortality, and cooccurring environmental events) influence the genetic architecture of resistance variants in modern populations [4]. The application of genomic sequence-based approaches to understand the host-pathogen interface continues to provide us with important clues for our survival. Human genetic variation is a major determinant of genetic susceptibility to many common infectious diseases [5]. Malaria, HIV/AIDS, and tuberculosis are some of the common infectious diseases in which a range of genetic susceptibilities and resistant conferring loci have been identified using both traditional molecular-based approaches and HTS technologies. HTS has enabled us to identify genomic signatures from these interactions. These markers identified through infectious disease genome-wide association studies (GWASs) have considerable significances that can be exploited to understand host protective mechanisms against pathogens and identify new molecular targets for diagnostic, prophylactic, and therapeutic interventions (Table 1).

In the past, candidate gene studies have been used to identify disease susceptibility genetic loci for many major human infections. But with the advent of HTS approaches, many new loci have been and continue to be identified in diverse populations. A paradigm shift from candidate gene studies to GWAS and HTS has ushered in a “big data” genomic revolution era enabling us to redefine the genomic architecture of host-pathogen disease susceptibility. Both exome and whole-genome sequencing approaches are proving more successful and affordable. Host genetics influence clinical course of infectious diseases as well as genetic variation of the pathogens determining their survival in presence of selective pressure from the host and environment (antimicrobials) and identify genetic markers of drug-resistant pathogens and parasites. Furthermore, HTS has given us unprecedented resolution of understanding the role of host genetics to infectious diseases susceptibility. This genomic revolution has generated data informative for understanding the frequency of many genetic traits, including those that cause disease susceptibility in African populations and populations of recent African descent [17].

**Figure 1:**
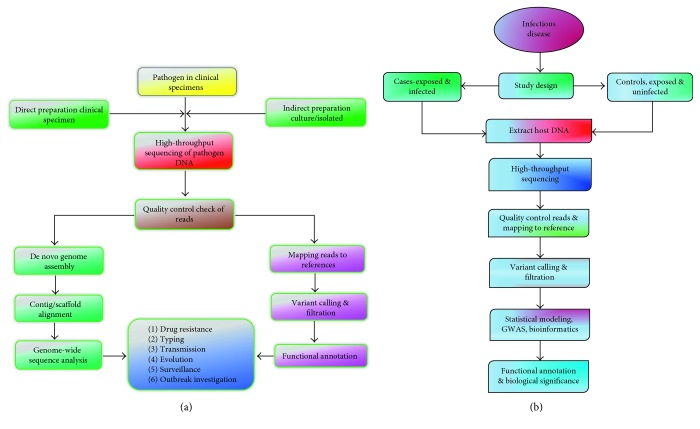
(a) Pipeline for interrogation of pathogen genomes using high-throughput sequencing and computational approaches. DNA extraction for HTS can be done from either direct clinical specimen of individuals who are suspected to be infected with the disease or from enriched/isolated cultures. Quality control and read preprocessing are critical steps in the analysis of datasets generated from high-throughput sequencing technologies. FASTQC is an example of a tool for general quality assessment of HTS data from all technologies. Genomes can be recreated with no prior knowledge using de novo sequence assembly as well as recreating the genome using prior knowledge based on a reference genome—alignment/mapping. The former is necessary for novel genomes and where the sequenced genome differs from reference. Sequence data analysis is important in infectious disease outbreak investigations, molecular typing, antimicrobial drug resistance, transmission, surveillance, and microbial evolution. (b) Pipeline for interrogation of host genomes using high-throughput sequencing and computational approaches. For a given infectious disease in a population, an appropriate study design is determined and host DNA is collected from cases (exposed to pathogen and infected) and controls (exposed to pathogen and uninfected). HTS of DNA from both cases and control is performed. Quality control (QC) procedures vary in different pipelines. These include QC on individuals for missingness, gender checks, duplicates and cryptic relatedness, population outliers, heterozygosity and inbreeding, QC on SNPs for missingness, minor allele frequency, and Hardy–Weinberg equilibrium. Many of these are computationally intensive, operationally challenging, and constantly evolving. Genome-wide association studies (GWASs) involving case-control studies compare the frequencies of common genetic variants, assume an appropriate statistical model, and account for multiple testing correction threshold to identify susceptibility and protective polymorphisms in the population.

Pathogens have always been a major cause of human mortality, so they impose strong selective pressure on the human genome [18, 19]. HTS applied to screening populations of host immune-specific cells and their respective pathogens can highlight the host-pathogen unique genetic signatures important in host-pathogen coevolution, profiling immunological history, pathogen-induced immunodominance genetic patterns, predicting clinical outcomes of common infections (such as HIV/AIDS disease progression phenotypes like long-term nonprogressors and rapid progressors, as well as highly exposed persistently seronegative group), rapid diagnosis plus screening outbreaks involving Risk Group 4 highly infectious pathogens, and genetic characterization of live-attenuated vaccine vectors (Figures 1(a) and 1(b)).

GWASs demand recruitment of large well-phenotyped clinical cohorts within appropriate study designs and settings. However, they are very expensive, and therefore few funding agencies are able to finance such studies yet they may offer unique opportunities to unveil genetically important signals. Currently, there are a growing number of communicable disease-specific research initiatives that are specifically interested in looking at the stages of the infections and GWAS of disease progression. A classic example is the Collaborative African Genomics Network (CAfGEN), a H3Africa-funded consortium probing host genetic factors that are important to the progression of HIV and HIV-TB infection in sub-Saharan African children (https://www.h3africa.org/consortium/projects/16-projects/89-cafgen). CAfGEN is specifically looking at both rapid and long-term HIV/AIDS progression status while utilizing a unique protocol that applies exome sequencing of AIDS extreme clinical phenotypes. The study designs and the protocols being utilized are valuable resources for future genomic research involving common infectious diseases. In this review, we describe the impact of HTS and genomics in understanding the human host-pathogen interaction.

## 2. Common Infectious Diseases

Infectious diseases account for 15 million deaths per year worldwide, and disproportionately affect the young, elderly people, and the poorest sections of society making them a high priority [20]. The World Health Organization in 2016 estimated the global mortality for tuberculosis, HIV/AIDS, and malaria to be at 3.23 million with most deaths occurring in sub-Saharan Africa. This region has continued to lead in both prevalence and incidence of these major infectious killer diseases [21]. Research investment in infectious diseases was poorly matched. Data show that funding does not correspond closely with burden [22]. Review of findings to date suggests that the genetic architecture of infectious disease susceptibility may be importantly different from that of noninfectious diseases [20]. Other authors have extensively reviewed the genetic susceptibility to diseases [4, 5, 13, 16, 18–20, 23–49]. The ancient biological “arms race” between microbial pathogens and humans has shaped genetic variation in modern populations, and this has important implications for the growing field of medical genomics [4]. As humans migrated throughout the world, populations encountered distinct pathogens, and natural selection increased the prevalence of alleles that are advantageous in the new ecosystems in both host and pathogens [4]. Temporal patterns supporting evidence of host-pathogen coevolution have been reported [50]. Common infectious diseases have shown geographical disparities, for example, *Mycobacterium tuberculosis* lineage 4 comprises globally distributed and geographically restricted sublineages [51]. Molecular epidemiological studies show that, with the exception of sub-Saharan Africa, almost all HIV-1 subtypes, circulating recombinant forms, and several unique recombinant forms have been detected, but there is a specific geographic distribution pattern for HIV-1 subtypes [52–54]. HIV diversity plays a central role in the HIV pandemic [55] and has significant implications for diagnosis, vaccine development, and clinical management of patients [56]. High levels of *Plasmodium falciparum* malaria endemicity are common in Africa [57]. Formal characterization of disease-causing agents has always been fundamental to understanding the evolution of pathogens and the epidemiology of infectious disease. Linking this information with temporal, spatial, and clinical data can bring understanding of evolution, geographical spread, and disease associations for the pathogen, providing vital information for identifying sources of infection as well as designing interventions to prevent and treat disease [58].

## 3. High-Throughput Sequencing (HTS) and Computational Tools

New sequencing technology has enabled the identification of thousands of single nucleotide polymorphisms in the exome, and many computational and statistical approaches to identify disease association signals have emerged [59]. Foremost is a better understanding of disease pathogenesis and resistance in the expectation that this will lead in time to improved interventions such as better drugs or vaccines to prevent or attenuate the great global burden of infectious disease morbidity and mortality. With over 10 million deaths annually from infectious diseases and the threat of new epidemics and pandemics, this is a very high priority [20]. During the past decade, we have also witnessed the emergence of many new pathogens not previously detected in humans, such as the avian influenza virus, severe acute respiratory syndrome (SARS), and Ebola [60]. Now that the scientific community has access to complete genomes of infectious diseases though application of HTS, priority should be the dissection of host-pathogen interactions through development of powerful computational tools. HTS has profoundly altered our understanding of human diversity and disease. The interaction between hosts and pathogens is a coevolution process which may simply be described as “shooting a moving target.” HTS and computational modelling tools will offer potential to understand this interaction leading to better vaccine designs and therapeutic targets. Sanger DNA sequencing is limited in throughput and high cost as compared to HTS platforms, which differ in their details but typically follow a similar general paradigm: template preparation, clonal amplification, followed by cyclical rounds of massively parallel sequencing [61]. Nanopore-based sequencing approaches such as single molecule, real-time (SMRT) sequencing technologies have been developed and consistently produce some of the longest average read lengths compared to HTS. SMRT sequencing is particularly useful for projects involving de novo assembly of small bacterial and viral genomes as well as large genome finishing [62]. Many important sequencing platforms are reviewed in great depth [61, 63].

Computational tools are an important integral part of genomics. New computational methods are constantly being developed to collect, process, and extract useful biological information from a variety of samples and complex datasets [64]. The scale and complexity of genomic data is ever-expanding, requiring biologists to apply increasingly more sophisticated computational tools in the generation, analyses, interpretation, and storage of this data. The data are generated in different sizes, formats, and structures requiring a wide range of tools to manipulate. Despite the level of specialization needed in bioinformatics, it is important that life-scientists have a good understanding of it for a correct experimental design which allows them to reveal the information in a metagenome [64]. HTS technologies are generating an astonishing amount of unprecedented information in the history of Biology which has spurred a Biomedical Big Data to Knowledge (B2D2K) revolution. Thus, a new exhilarating rapidly evolving scientific field, Bioinformatics (Biology meets computer programming), has recently emerged and uses novel computational approaches to help solve important biological problems. Bioinformatics is a set of activities: data acquisition, database development, data analysis, data integration, and analysis of integrated data. The majority of available bioinformatics software requires some knowledge of the text-based command line of the UNIX or Linux operating systems, allowing custom programming scripts and pipelines to automate data manipulation and analysis in a single step [65]. Although bioinformatics tools/software are both “open sources” and commercially available, clinicians have limited bioinformatics knowledge [66–69]. It is clear that user-friendly bioinformatics pipelines are key to facilitating more widespread use of WGS, with more widespread bioinformatics expertise [65].

## 4. Signatures of Selection on the Genomes

Positive selection (also known as Darwinian selection) in genes and genomes can point to the evolutionary basis for differences among species and among races within a species [70]. Many other aspects of human biology not necessarily related to the “branding” of our species, for instance, host-pathogen interactions, reproduction, dietary adaptation, and physical appearance, have also been the substrate of varying levels of positive selection. Comparative genetics/genomics studies in recent years have uncovered a growing list of genes that might have experienced positive selection during the evolution of humans and/or primates [71]. Microbial genome evolution is shaped by a variety of selective pressures. Understanding how these processes occur can help to address important problems in microbiology by explaining observed differences in phenotypes, including virulence and resistance to antibiotics. Greater access to whole-genome sequencing provides microbiologists with the opportunity to perform large-scale analyses of selection in novel settings such as within individual hosts [72].

Identification of positive selection genetic signatures in the genomes can help us to understand the kinetics and directions of continuing host-pathogen coadaptation and impact on their diagnosis, transmission, fitness, immunogenicity, and pathogenicity. Given the potential for strong selective pressure, that genetic programs controlling host-pathogen interactions in humans and other species are littered with signatures of positive selection [73]. The high mortality and widespread impact of malaria has resulted in this disease being the strongest evolutionary selective force in recent human history, and the genes that confer resistance to malaria provide some of the best-known case studies of strong positive selection in modern humans [27]. A number of specific genomic variants including *β*-globin locus, G6PD deficiency, Duffy, ovalocytosis, ABO, and human leukocyte antigen confer resistance to malaria in the human host. Elevated frequencies of hemoglobinopathies such as thalassemia and sickle cell disease, which are caused by mutations at the *β*-globin locus, are maintained via balancing selection (“the malarial hypothesis”) [74–76]. This hypothesis suggests that some human diseases such as thalassemia are polymorphisms which provide heterozygote advantage because of the trade-offs between the advantages of resistance to malaria and negative effects due to the disease [77], where the hemoglobin S (HbS) homozygote disadvantage is recompensed through the malaria resistance of the heterozygote (HbAS) in regions of malaria endemicity [78, 79]. The ∆32 mutation at the CCR5 locus is a well-studied example of natural selection acting in humans. The mutation is found principally in Europe and western Asia, with higher frequencies generally in the north. Homozygous carriers of the ∆32 mutation are resistant to HIV-1 infection because the mutation prevents functional expression of the CCR5 chemokine receptor normally used by HIV-1 to enter CD4+ T cells [80–87]. Host genetic factors play important roles in susceptibility to tuberculosis infection, and different gene polymorphisms in different ethnicity and genetic backgrounds may lead to different effects on tuberculosis risk [88]. Polymorphisms in natural resistance-associated macrophage protein 1 (NRAMP1), toll-like receptor 2 (TLR2), interleukin-6 (IL-6), tumor necrosis factor-alpha (TNF-*α*), interleukin-1 receptor antagonist (IL-1RA), IL-10, vitamin D receptor (VDR), dendritic cell-specific ICAM-3-grabbing nonintegrin (DC-SIGN), monocyte chemoattractant protein-1 (MCP-1), nucleotide oligomerization binding domain 2 (NOD2), interferon-gamma (IFN-*γ*), inducible nitric oxide synthase (iNOS), mannose-binding lectin (MBL), and surfactant proteins A (SP-As) genes have been variably associated with tuberculosis infection, and there is strong evidence indicating that host genetic factors play critical roles in tuberculosis susceptibility, severity, and development among different populations [89–91]. Several *NRAMP1* polymorphisms were significantly associated with PTB in African and Asian populations, but not in populations of European descent [92, 93].

## 5. Genome-Wide Association Studies in Infectious Diseases

GWASs are based on the “common disease, common variant” hypothesis, and they have been performed largely using single nucleotide polymorphism (SNP) arrays that focus only on common genetic polymorphisms (for which the minor allele frequency is >5%) [35, 94–96]. GWAS approach has potential to provide candidates for the development of control measures against infectious diseases in humans [13]. For over 50 years, candidate gene studies have been used to identify loci for many major causes of human infectious mortality, including malaria, tuberculosis, and HIV-1 [20]. The first successful GWAS was published in 2005 ushering genome-wide approaches that have identified loci in diverse populations. Common genetic variants have also been demonstrated to regulate susceptibility/resistance to infectious diseases, for example, the *CCR5*∆32 polymorphism that modulates HIV/AIDS disease progression [97]. Genome-wide association study approaches are being increasingly utilized to define genetic variants underlying susceptibility to major infectious diseases [34]. Infectious diseases follow a series of stages right from acquisition, disease development, rate of progression, convalescence, and asymptomatic carrier state. Therefore, every stage will be influenced by one or a set of mutations in a population or individual. Different populations will have mutations that affect diseases at different stages. This is where GWAS has and will continue to play an important role in identifying these mutations. A recent study suggested that host genetic risk in TB is depended upon the pathogen's genetic background and demonstrated the importance of analyzing the interaction between host and pathogen genomes in TB [98]. Studies are exploiting unique designs like extreme phenotype designs to identify complex trait genomic loci, while others have identified genetic associations of infectious diseases by integrating estimation of population admixture events to detect disease susceptibility loci after teasing out different ancestries and allelic, genotypic, or haplotype risk ratios.

**Figure 2:**
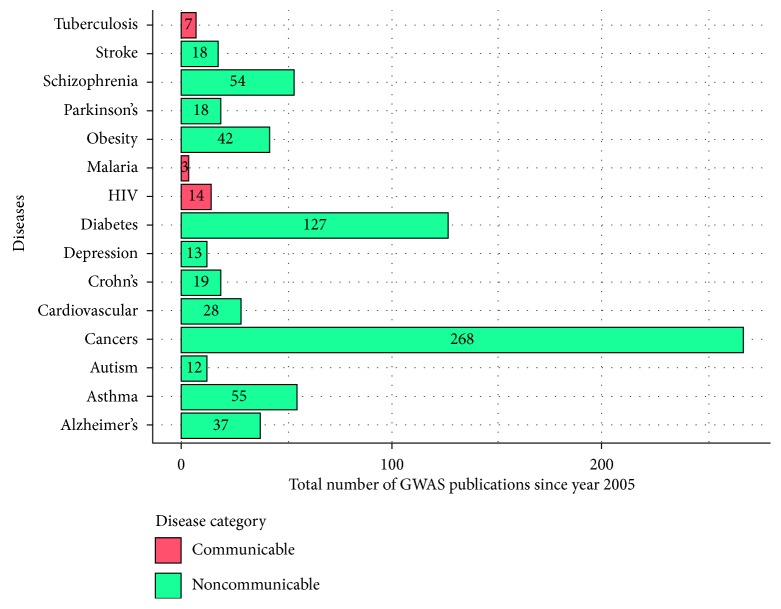
Comparison of selected communicable and noncommunicable disease GWASs since 2005.

A genomic database for all mutations identified to be conferring resistance to infectious diseases in different populations is a vital product of more than 10 years of GWAS. More studies should be appropriately designed to identify new potential infectious disease resistance-conferring mutations in human hosts. We searched the PubMed database for studies published since January 2005 using the terms “malaria,” “tuberculosis,” “HIV/AIDS,” “genome-wide association.” Search terms included combinations of ((*disease-query*[Title] AND Genome-wide association[Title])) where a disease-query was either a communicable or noncommunicable disease. Two search restrictions were set: publication date set (from 2005/01/01) and species (humans).  Figure 2 indicates more GWASs carried out in noncommunicable diseases than infectious diseases. There is an urgent need for a major increase in funding for communicable disease control in the developing world and for more balanced allocation of the resources already provided [22].

## 6. Future Directions

Genomics and whole-genome sequencing have the capacity to greatly enhance knowledge and understanding of infectious diseases and clinical microbiology [65]. Human genetics is an indispensable tool for enhancing the understanding of the molecular basis of many common diseases [99]. Over 4,500 SNPs have been associated with a variety of human traits and complex diseases [59]. We envisage that with the reducing costs, HTS and genomics will become an indispensable component of every health-care system. HTS and computational tools offer a potential to stratify populations for risk of infectious disease based on genomic profiling thereby prioritizing interventions such as vaccines and therapeutics to the “most-at-risk” populations since there is no “one-size-fits-all” approach to treating infectious diseases. With the growing number of sequencing facilities on every continent, the future will offer a less costly approach that will integrate a genomic profile in routine patient management, improving management of diseases, and therapeutic development.

It is less likely to find a population or individual who carries mutations conferring resistance to an infectious disease at every stage of the infection. Different individuals have different mutations that offer resistance to different stages of the infections. A genomic catalogue of these mutations identified through HTS, computational tools, and GWAS now combined with the rapidly growing genome-editing technology known as CRISPR/Cas9 will enable introduction of an array of disease-stage-specific resistance-conferring mutations. This will offer interventions at all levels of the disease process unlike traditional vaccines.

## Figures and Tables

**Table 1:** Host loci strongly associated with the three common infectious diseases.

Disease	Population	Variant	Annotation	Genome-wide significance (*P* value)	Association	References
HIV-1/AIDS	African Americans	rs2523608	*HLA-B^∗^5703*	5.6 × 10^−10^	Viral load set point	[6]
	European ancestry	rs2395029	*HCP5/B^∗^5701*	9.7 × 10^−26^	Long-term nonprogression and viral load set point	[7–9]
	European ancestry	rs9264942	*HLA-C*	2.8 × 10^−35^	Long-term nonprogression and viral load set point	[7]
	European ancestry	rs4418214	*MICA*	1.4 × 10^−34^	Long-term nonprogression	[7]
	European ancestry	rs3131018	*PSORS1C3*	4.2 × 10^−16^	Long-term nonprogression	
	African American	rs2255221	*Intergenic*	3.5 × 10^−14^	Long-term nonprogression	
		rs2523590	*HLA-B*	1.7 × 10^−13^	Long-term nonprogression	
		rs9262632	*Intergenic*	1.0 × 10^−8^	Long-term nonprogression	
	European ancestry	rs9261174	*ZNRD1*, *RNF39*	1.8 × 10^–8^	Disease progression	[8, 10]
		rs11884476	*PARD3B*	3.4 × 10^–9^	Disease progression	[11]
		rs2234358	*CXCR6*	9.7 × 10^–10^	Long-term nonprogression	[12]
Malaria	Ghanaian	rs10900585	*ATP2B4*	6.1 × 10^−9^	Protective	[13]
	Ghanaian	rs2334880	*MARVELD3*	3.9 × 10^−8^	Protective	[13]
	Ghanaian	rs8176719	*ABO*	2.9 × 10^−13^	Protective	[13]
	Gambian	rs11036238	*HBB*	3.7 × 10^–11^	Susceptibility	[14]
	Gambian	rs334	*HBB*	4 × 10^−14^	Protective	[13, 14]
Tuberculosis	Moroccan	rs17590261	*AGMO*	2 × 10^−6^	Age-at-onset of TB	[15]
	Ghanaian and Gambian	rs4331426	18q11.2	6.8 × 10^−9^	Susceptibility	[16]
